# From Neural and Social Cooperation to the Global Emergence of Cognition

**DOI:** 10.3389/fbioe.2015.00078

**Published:** 2015-06-16

**Authors:** Paolo Grigolini, Nicola Piccinini, Adam Svenkeson, Pensri Pramukkul, David Lambert, Bruce J. West

**Affiliations:** ^1^Center for Non-linear Science, Department of Physics, University of North Texas, Denton, TX, USA; ^2^Army Research Laboratory, Adelphi, MD, USA; ^3^Faculty of Science and Technology, Chiang Mai Rajabhat University, Chiang Mai, Thailand; ^4^Information Science Directorate, US Army Research Office, Research Triangle Park, NC, USA

**Keywords:** temporal complexity, phase transition, criticality-induced synchronization, game theory, behavioral psychology, neural and social criticality

## Abstract

The recent article (Turalska et al., 2012) discusses the emergence of intelligence via criticality as a consequence of locality breakdown. Herein, we use criticality for the foundation of a novel generation of game theory making the local interaction between players yield long-range effects. We first establish that criticality is not confined to the Ising-like structure of the sociological model of (Turalska et al., 2012), called the decision making model (DMM), through the study of the emergence of altruism using the altruism-selfishness model (ASM). Both models generate criticality, one by imitation of opinion (DMM) and the other by imitation of behavior (ASM). The dynamics of a sociological network 𝒮 influences the behavioral network ℱ through two game theoretic paradigms: (i) *the value of altruism*; (ii) *the benefit of rapid consensus*. In (i), the network 𝒮 debates the moral issue of altruism by means of the DMM, while at the level ℱ the individuals operate according to the ASM. The individuals of the level 𝒮, through a weak influence on the individuals of the level ℱ, exert a societal control on ℱ, fitting the principle of complexity management and complexity matching. In (ii), the benefit to society is the rapid attainment of consensus in the 𝒮 level. The agents of the level ℱ operate according to the prisoner’s dilemma prescription, with the defectors acting as DMM contrarians at the level 𝒮. The contrarians, acting as the inhibitory links of neural networks, exert on society the same beneficial effect of maintaining the criticality-induced resilience that they generate in neural networks. The conflict between personal and social benefit makes the networks evolve toward criticality. Finally, we show that the theory of this article is compatible with recent discoveries in the burgeoning field of social neuroscience.

## 1 Introduction

The recent article (Turalska et al., 2012) offers a new theoretical perspective on the network science of learning that we adopt herein to explain the emergence of altruism and cognition in complex networks. The emergence of altruism seems to conflict with the widely accepted idea that the action of the individual is mainly dictated by the goal of maximizing personal profit. One purpose of game theory is to determine the truth of this conviction. This too is the main goal of the new forms of game theory that we propose herein. The novelty of the present approach is the adoption of concepts developed in the field of complex networks and especially those from the last frontier of complexity (D’Agostino and Scala, 2014), namely, from the subject of multilayer networks. To explain the significance of the proposed approach, it is necessary to compare it with the dominant views from the field of complex networks.

The theory of complex networks has been shaped, in part, by the pioneering work of Albert and Barabási (2002) who popularized the class of complex networks whose probability density function (PDF) *P*(*k*) of links has an inverse power-law structure

(1) $$P\left(k\right)∼\frac{1}{k^{\gamma }}.$$

Due to the fact that this PDF describes a network having multiple scales, with no one scale dominating, it is referred to as a *scale-free distribution of links* and the network is called scale-free. This is the origin of the popularity of complex networks, interpreted as being characterized by a scale-free distribution of links. This success led many researchers to study dynamical processes occurring in such scale-free networks within multiple disciplines. In the original model of Albert and Barabási, a scale-free network is generated by a dynamical process in which new nodes are attracted by previously existing nodes in proportion to the number of links to those nodes. We are convinced that what has been learned from the study of dynamical processes on scale-free networks has been based on the implicit assumption that the process responsible for the emergence of a scale-free PDF of links has been exhausted. Thus, those studies have been limited to the benefits emerging from the adoption of a scale-free topology, with little or no discussion of the open problem of what sort of dynamics occurs while the scale-free distribution of links is taking shape.

Turalska et al. (2012) show that a scale-free topology can emerge from an ordinary regular lattice, where, in accordance with the principle that *all men are created equal*, each of the individuals in the network have the same number of links. Every organized “society,” ranging from human (Turalska et al., 2013) to neural (Fraiman et al., 2009), is assumed to be at criticality. The dynamics of criticality generate time-dependent links with scale-free PDFs (Fraiman et al., 2009; Turalska et al., 2012). Thus, we have a few individuals with a large number of links and many more individuals with only a few links, a condition that is dynamic rather than static. It has been determined that leadership in such networks changes from one individual to another and that in the long-time dynamics the leadership role is uniformly shared by all individuals within the network.

The adoption of a static scale-free topology prevents one from discovering another important property emerging from Turalska et al. (2012): criticality-induced long-range correlation. This emergent correlation makes it possible to go beyond the local nature of the current forms of game theory (Nowak and May, 1992, 1993; Eshel et al., 1998). Embedding a scale-free network into a two-dimensional lattice has the effect of generating large-scale perception (Hollingshad et al., 2012), thereby reiterating the importance of criticality-induced long-range correlation. Herein, we use the dynamic properties of complex networks (Turalska et al., 2012) to develop new forms of game theory and a new interpretation of cognition as well. We interpret the emergence of cognition as an effect produced by the transmission of information from one complex network at criticality to another complex network at criticality (Vanni et al., 2011; Luković et al., 2014).

In Section 2, we discuss a model for the imitation-induced emergence of altruism. We show that this altruism model shares with the cooperative models of earlier work (Vanni et al., 2011), the property of generating long-range correlation between distant elements, an important property that we plan to emphasize in the new forms of game theory. Section 3 explains that the two forms of game theory we propose in this article are based on the interaction between the sociological network 𝒮 and the behavioral network ℱ. Section 4 illustrates the *value of altruism* game theory. Section 5 illustrates the *benefit of rapid consensus* game theory. In Section 6, we argue that our approach to game theory may shed light on the emergence of cognition and facilitate progress toward a behavioral approach to economics.

## 2 Dynamics of Altruism and Selfishness

The model we study in this section is based on two states: *A* denoting altruism and *S* selfishness. The main purpose of this section is to establish that, due to imitation-induced criticality, an action exerted on a small group of individuals localized on a two-dimensional regular network, is transmitted to long distances from this small group. This transmission of information supports our claim that the behavior of rewarding fairness or punishing selfishness even if applied locally to a specific set of individuals may have long-range effects. This would be assured if the game theory is designed in such a way as to include criticality and temporal complexity (Turalska et al., 2011).

Following Turalska et al. (2012), we assumed a two-dimensional lattice, and assign to each individual located on a node either the state *A* or the state *S*, with random initial prescription. At each time step, the elements can either change state or remain in the same state, according to the following procedure. If the element is in the state *A*, it can jump to the state *S* with an exponential PDF at the rate

(2) $$\gamma =\gamma_{0}-\beta \frac{M_{A}}{M},$$

where *M* is the number of nearest neighbors of the element and *M_A_* the number of nearest neighbors in the state *A*. We use a two-dimensional lattice with *M* = 4. This prescription implies that if the individual is surrounded by only selfish people s/he has a tendency to move to the selfish state as a consequence of human nature. Here, human nature is assumed to be non-altruistic. However, if some of the neighbors are altruistic, the transition rate decreases. If the individual is in the state *S*, the transition rate is given by

(3) $$\omega =K\frac{M_{A}}{M}.$$

**Figure 1:**
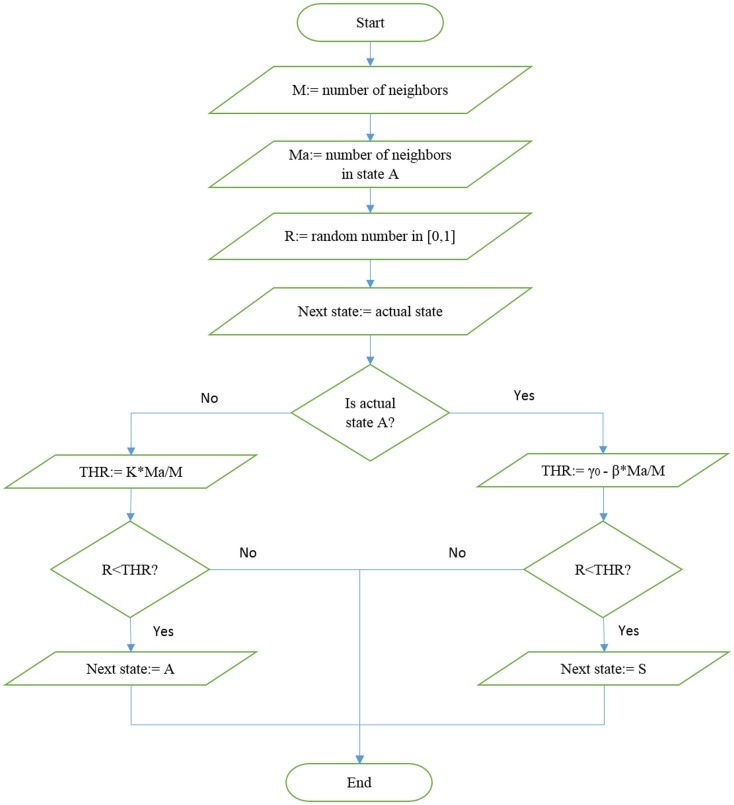
**Illustration of the algorithm used to update the state in the altruism-selfishness model**.

In this case, no transition occurs if all the neighbors are selfish. The algorithm generating the choice of either selfishness or altruism is illustrated in Figure 1. The network is expected to reach criticality, namely achieve a condition where altruism does not become extinct, by either increasing *β* or increasing *K*.

**Figure 2:**
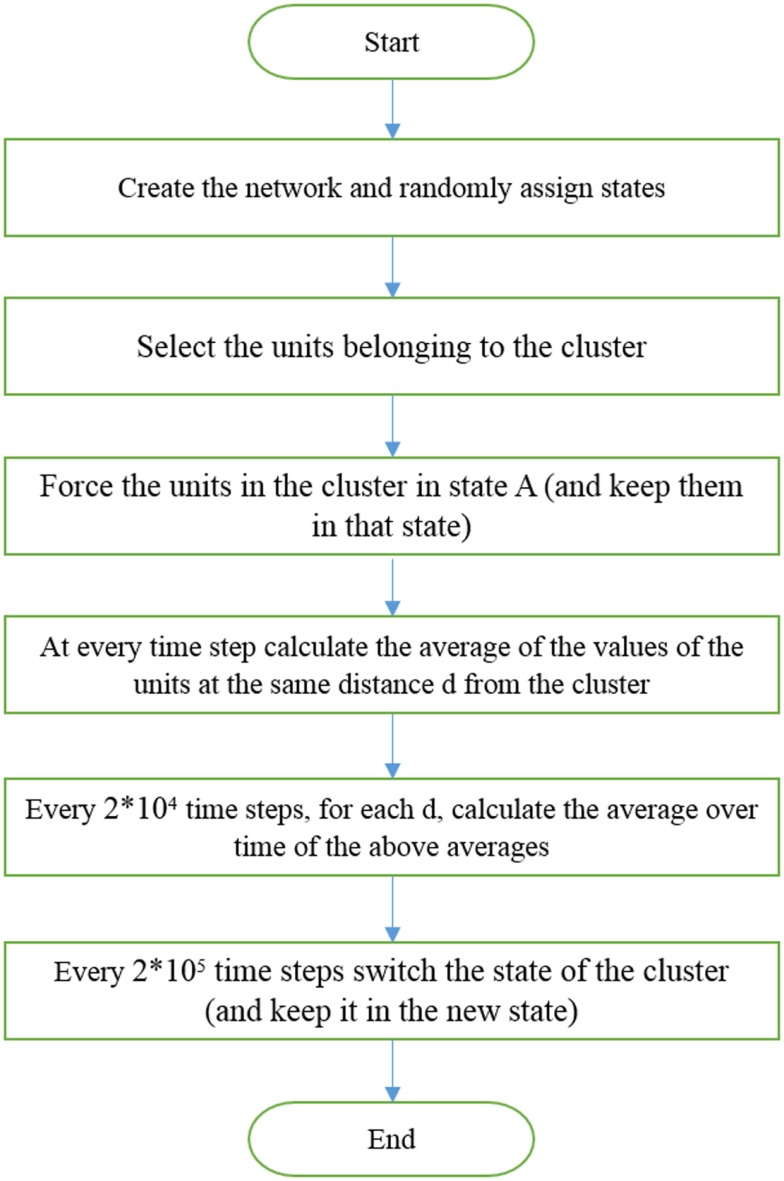
**Illustration of the algorithm used to generate Figure 3**.

Here, we keep *K* fixed and generate criticality by changing *β*. After randomly distributing the individuals on the network, half in each state, the model is run for a total time *T_total_* = 1.01 × 10^8^. We externally force a small cluster, 1% of the total number of individuals, to adopt the altruist state for a time *T* = 2 × 10^5^. Then, we externally force this same cluster into the selfishness state for a time interval of equal length. The time evolution of all the elements at distance *d* from those transmitting the dichotomous signal is monitored. We divide the interval *T* into smaller time intervals of equal size 2 × 10^4^. In each of these smaller intervals, the single elements have fluctuations that are averaged over so that each subinterval is assigned a value equal to the time average. Subsequently, the average is taken over the elements at the same distance *d* from the driving group. The algorithm used to force the whole system to adopt either altruism or selfishness, via criticality-induced long-range correlation, is illustrated in Figure 2.

**Figure 3:**
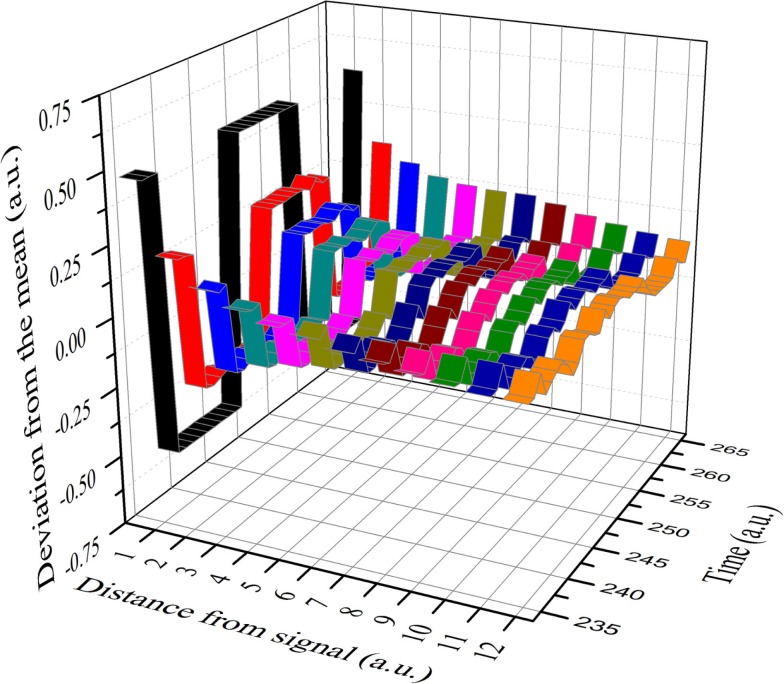
**A small cluster (1% of the total number of units) is forced to stay in the altruism state for a time T, then to move to the selfishness state for the same time T, and so on in this manner**.

**Figure 4:**
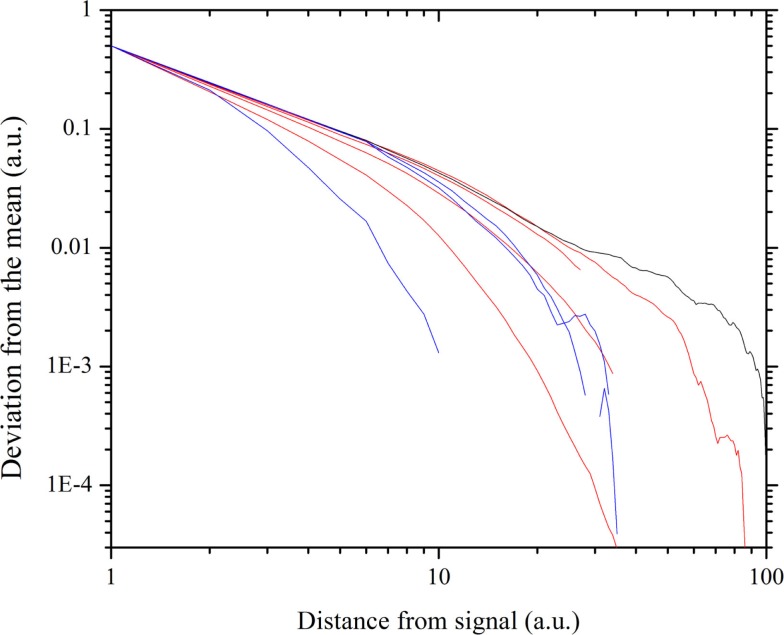
**Decay of the information with the distance, obtained by recording the top envelope**. The gray straight line, with power-law index 1.28, fits the thick black line, corresponding to the critical value *β* = 0.033. The red curves correspond to the *β* values (approaching criticality) 0.0, 0.02, 0.027, 0.03. The blue curves correspond to the *β* values (departing from criticality) 0.036, 0.04, 0.08.

The result of this numerical procedure is illustrated in Figure 3. The dichotomous black line is the signal that the small cluster of 1% of the total number of units transmits to all the other units of the same network. Figure 3 shows that the information of the transmitted signal becomes less and less accurate as the distance between the transmitters and the receivers increases. In other words, this is the same as the numerical experiments done previously (Vanni et al., 2011; Luković et al., 2014), with the added benefit of a more quantitative analysis of the criticality-induced enhancement of information transmission. In Figure 3, we see that the intensity of the signal diminishes as the distance between the driven elements and those responding via the network’s dynamics increases. Note that below and above criticality, the signal decay is exponential, while at criticality, it is an inverse power-law. Figure 4 shows this important property in detail. Moving the altruism parameter from *β* = 0 to *β* = 0.033, the information decay is exponential with a decreasing rate. For values of *β*, increasingly departing from criticality, the information decay becomes faster and faster. At criticality, *β* = *β*_c_ = 0.033, we fit the information decay with an inverse power-law with power index ν = 1.28.

In Section 4, we discuss how to use this imitation approach to altruism for the first form of game theory proposed herein. The results of this section, in accordance with the views of West et al. (2014), confirm the sociological importance of imitation and afford support to the goal of this article: establishing a new form of game theory taking imitation into account.

## 3 Toward a New Game Theory

The prisoner’s dilemma was invented to codify and investigate the conflict between cooperation and personal benefit in human decision making. In their game theory, Nowak and May (1992) adopted the prisoner’s dilemma to address the challenging issue of explaining how cooperation may emerge from the social interaction of individuals who are assumed to make decisions on the basis of maximizing personal profit. Another version of game theory includes the concept of cooperation cost, introduced to explain why cooperation may enhance the overall payoff without eliminating the incentive to defect (Szabó and Fáth, 2007). According to social psychology, the decision-making process of individuals is not only determined by the criterion of maximal financial benefit, but also depends on emotions, intentions, and beliefs that influence the interaction of one individual with others. In this way, social behavior can have a significant neurophysiological origin (Frith, 2007; Declerck et al., 2013; Bhanji and Delgado, 2014; Tognoli and Kelso, 2014). Experimental social psychology supports this view: for instance, Kearns and co-workers (Kearns et al., 2006, 2009; Judd et al., 2010; Kearns, 2012) study the dynamics of a group of individuals, who are assigned the task of reaching consensus with the payoff being determined by the successful achievement of consensus.

This juxtaposition of the social with the psychological suggests a connection with the subject, currently in a phase of rapidly increasing scientific interest, of multilayer networks (D’Agostino and Scala, 2014). This connection need not have the focus on failure suggested by the pioneering paper (Buldyrev et al., 2010). The studies (Boccaletti et al., 2014; Kivelä et al., 2014) moving in these challenging directions seem to focus on the topology of the links connecting different layers. The sociological and the economic levels of this article, on the contrary, are two distinct networks with the simple lattice topology of a two-dimensional regular network, interacting at criticality. Since criticality generates an effective network of strongly correlated elements with the structure of a scale-free network (Turalska et al., 2012), these layers can be visualized as inter-linked scale-free systems, with a criticality-generated complex topology. Although the presented approach rests on a prescription valid for any topology, running it on a scale-free network would have inhibited our ability to appreciate the long-range correlations generated by criticality (Turalska et al., 2012). Using this insight, we propose a new form of game theory defined on a regular two-dimensional network.

**Figure 5:**
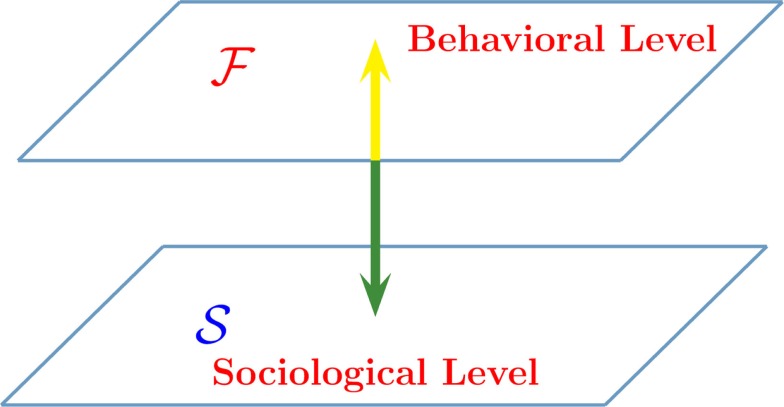
**Interaction between two complex dynamic networks**. The network 𝒮 is a two-dimensional regular lattice devoted to either a moral debate (the value of altruism) or to making decision on some issue of social interest (the benefit of rapid consensus). The network ℱ is the corresponding network where the single individuals act on the basis of either their personal financial benefit (the benefit of rapid consensus), or as a result of their interaction with the nearest neighbors (the value of altruism).

Figure 5 illustrates how the new game theory is formulated. The level 𝒮 is the sociological–psychological level and for simplicity, we assume that the elements of this network are driven by the decision making model (DMM). We propose two distinct forms of game theory: (i) *the value of altruism*; (ii) *the benefit of rapid consensus*. In the (i) form of game theory, the sociological debate, occurring in the 𝒮 level, concerns whether it is convenient to adopt the state *A* or the state 𝒮. In the ℱ level, the single individual acts on the basis of their personal inclination and also as a consequence of their imitation of the behavior of their neighbors. In the (ii) form of game theory, the sociological debate also occurs in the 𝒮 level. However, in this case, the benefit for society is the expeditious attainment of consensus. The same individuals, operating in the level ℱ are governed by a conventional form of game theory where they are driven by the goal of maximal financial benefit. This explains why we adopt the symbol ℱ to denote this level. However, to be consistent with the spirit of the (i) game theory, we refer to the level ℱ as the *behavioral level*.

## 4 Evolutionary Game Theory: The Value of Altruism

The model of Section 2 can generate the spreading of selfish individuals over the whole network if the parameters *β* and *K* are sufficiently small. However, the excessive proliferation of selfish individuals can be kept under control by the sociological level 𝒮. This approach to the containment of selfishness is done according to the spirit of the work (Piccinini et al., in preparation), which is devoted to the societal control of the spreading of a sexually contracted infection. Piccinini et al. (in preparation) study the influence of a societal debate on whether to adopt safe sex practices or not, and the proposed evolutionary game theory presented herein becomes virtually equivalent to the complex dynamics studied by them (Piccinini et al., in preparation). This equivalence requires that the altruist selecting the state *A* is identified with the safe sex user, and the individual opposing the adoption of safe sex precautions is identified with the individual selecting selfishness, state *S*. In the situation they (Piccinini et al., in preparation) study, the sociological debate may favor the state *A* because the choice of the state *S* is connected to the spreading of an infectious disease.

The sociological debate in the case of *the value of altruism* game refers to the choice between the state *A* and the state *S* of Section 2. We make the assumption that the individuals of the sociological level 𝒮 debate the social value of altruism and the dangers of selfishness.

The connection between criticality and game theory is realized on the basis of the societal value assigned to the states *A* and *B*. In this version of game theory, the level 𝒮 is driven by the DMM at its critical point, while the ℱ level is driven by the two-state model described in Section 2, with *β* = 0.003 and *K* = 0.015, namely, also at its critical point. The system ℱ, in the absence of coupling with the system *S*, has a mean field y=A−Ā, which is characterized by temporal complexity (Turalska et al., 2011).

Notice that the level 𝒮 drives the level ℱ by means of very weak coupling. If a given element at the level 𝒮 is in the state *A* favoring the altruistic choice, then the parameters *β* and *K* are incrementally increased in such a way as to increase the lifetime of the altruism state in level ℱ. As a consequence, the correlation function, with y=A−Ā and $x=B-\bar{B}$

(4) $$C\left(t\right)=\frac{\left\langle x\left(t\right)y\left(t\right)\right\rangle }{{\left\langle x^{2}\right\rangle }^{1∕2}{\left\langle y^{2}\right\rangle }^{1∕2}}$$

is expected to fit the predictions of complexity management (Aquino et al., 2010). This situation leads to the correlation attaining its maximum, which is an important case discussed in detail elsewhere. Here, we limit ourselves to pointing out two significant aspects of this form of evolutionary game theory: (1) *the emergence of long-range correlation* and (2) *criticality-induced transmission of information*.

### 4.1 Long-Range Effects

Such effects are present in both the sociological and behavioral levels. As explained in Section 2, if some individuals on the level ℱ are forced to adopt a given dependence on time, this behavior is transmitted to all the other individuals of the network. On the one hand, this property had been previously discussed (Vanni et al., 2011; Luković et al., 2014). On the other hand, if the sociological level operates at criticality, we meet the long-range correlation of the even earlier work (Turalska et al., 2012).

### 4.2 Information Transmission

To properly appreciate the contribution of this two-layer network to the laying of a solid scientific foundation for the widely accepted conviction that criticality favors information transmission (Turalska et al., 2009; Mora and Bialek, 2011; Attanasi et al., 2014; Hidalgo et al., 2014), we stress the special role played by temporal complexity (Turalska et al., 2011). Mora and Bialek (2011) recently remarked that the onset of a phase transition implies *critical slowing down*, and consequently an extremely slow regression of a network perturbation to equilibrium. This slowing down seemed to them to be incompatible with, for instance, the expected rapid response of a flock of birds to a predator. The theoretical perspective adopted herein establishes a clear distinction between critical slowing down and temporal complexity: in spite of both being generated by criticality, these are two distinct properties of dynamics (Bologna et al., 2013).

Temporal complexity is a property of phase transition processes which occurs when the network has a finite number of interacting individuals, and which vanishes in the thermodynamic limit, i.e., when the network size becomes infinite. Turalska et al. (2009) show that there is a close connection between temporal complexity and ergodicity breakdown. Criticality-induced fluctuations are renewal non-Poisson properties (Bianco et al., 2008) characterized by the phenomenon of renewal aging, and so by a condition physically equivalent to an extended sojourn in an out-of-equilibrium state. In the last few years, some fundamental work has been done to establish the response of these complex networks to external perturbation (Barbi et al., 2005; Allegrini et al., 2007), resulting in a linear response theory that led to a remarkably accurate agreement with experiment (Allegrini et al., 2009; Silvestri et al., 2009). The experimental preparation of non-ergodic complex networks generates a non-stationary cascade of events, thereby making these systems insensitive to perturbations that do not share their complexity. This observation led to the important principle of complexity management (Aquino et al., 2010), namely, a steady correlation of complex fluctuations with stimuli of equal or higher complexity, revealed by the method of ensemble averages. The phenomenon of complexity matching (Turalska et al., 2009) has the same origin as complexity management, but it is revealed by single realizations and is proved (Luković et al., 2014) to generate synchronization between two criticality-induced fluctuations in the course of their regression to equilibrium after proper preparation. In conclusion, criticality-induced synchronization between two complex networks is a consequence of temporal complexity and of the related aging process. Complexity matching (Zare and Grigolini, 2013) is realized (Luković et al., 2014) as a form of response of a complex network at criticality to the stimulus exerted on it by another complex network at criticality, both being far from the thermodynamical limit.

It is very important to stress that criticality-induced temporal complexity is associated with ergodicity breakdown, this being a subject of great interest in the field of molecular diffusion in biological cells (Metzler et al., 2014). The discovery of ergodicity breakdown is one of the most important scientific results generated by 100 years of single particle tracking (Metzler et al., 2014) and is forcing researchers in this field to go beyond the limits of conventional statistical physical thought. The study of sociological and neurophysiological networks is based on the observation of single systems, an ensemble average over copies of the same brain, for example, being senseless. For a statistical analysis of these systems, one is compelled to adopt the procedures that are emerging from the field of molecular diffusion in biological cells. The adoption of the time averages made necessary by the fact that only one sociological trajectory is available led to the discovery (Piccinini et al., in preparation) of ergodicity breakdown in sociology as well as neurophysiology (Turalska et al., 2009). Establishing whether sharing ergodicity breakdown implies that sociological, neurophysiological, and biological systems are all in an out-of-equilibrium state is an open and stimulating problem. While expressing our wish that this article may attract the attention of researchers on this fundamental issue, we stress that complexity management and complexity matching processes go far beyond the pioneering work of Trefán et al. (1994) and the more recent work of Godec and Metzler (2013). In fact, this earlier work deals with the response to perturbation of Lévy walk diffusion processes, a case in which ergodicity breakdown and aging are temporary and not perennial as in the case of systems responding to perturbation according to the predictions of complexity management (Aquino et al., 2010) and complexity matching (Turalska et al., 2009; Luković et al., 2014).

## 5 Evolutionary Game Theory: The Benefit of Rapid Consensus

This section is devoted to illustrating *the benefit of rapid consensus* game. According to Standard and Poor’s, the October 2013 shutdown of the Federal Government of the United States of America took 24 billion dollars out of the U.S. economy, and reduced the projected fourth-quarter GDP growth from 3 percent to 2.4 percent. In the face of such economic swings, it is plausible to conjecture that the rapid attainment of consensus is beneficial for the society as a whole, and that eventually consensus can be measured in terms of monetary gain. Therefore, reaching consensus on level 𝒮 is a condition that is incorporated into the proposed game theory model. The results of psychological experiments (Kearns, 2012) show that consensus is reached through the *local interactions* of individuals. This important property is inexplicable at first sight. In fact, it seems to be impossible that a social network reaches consensus without the condition that all the individuals interact with all the other individuals, the All-To-All (ATA) condition.

The network 𝒮 exerts a constraint on the network ℱ based on the assumption that reaching consensus is very important for society to function. The DMM is a theoretical sociological model based on the local imitative interaction of individuals that nevertheless, when the inter-individual coupling is strong enough, generates global properties (West, 2011). This cooperative global behavior occurs because a sufficiently strong local interaction can generate a phase transition. At the onset of a phase transition, that is to say, at *criticality*, the correlation length between individuals becomes as large as the size of the social network (Turalska et al., 2012). In other words, at criticality, a social network functioning on the basis of local interactions, becomes indistinguishable from an ATA network. As a consequence of this observation, the dynamics of level 𝒮 is based on the DMM (West, 2011). Alternatively, it may be based on the swarm model (Vicsek et al., 1995), established in recent work (Vanni et al., 2011) to be characterized by criticality. Criticality turns the modeled social network into an ATA network, in spite of the local character of the interaction between its elements (Vanni et al., 2011).

In summary, the dynamics of level 𝒮 is based on local interactions, in the spirit of game theory, but at the same time, the new approach fits recent discoveries in the field of behavioral psychology concerning social influence on the decision-making process by single individuals (Frith, 2007) and, thanks to criticality, explains why social cooperation at the local level may accomplish global tasks (Kearns et al., 2006, 2009; Judd et al., 2010; Kearns, 2012).

### 5.1 Dynamics of level ℱ

The hereby illustrated preliminary results refer to the prisoner’s dilemma scenario. In this game, a cooperator playing with another cooperator receives a reward of 1, thereby yielding a financial gain of 2 for society as a whole. A defector playing with a cooperator receives *b* > 1, while the cooperator receives 0, this being an incentive to defect. A defector playing with another defector receives 0. The social dilemma in this case is given by the condition

(5) 1<b<2.

In fact, the condition *b* < 2 corresponds to affording to the whole of society a financial benefit smaller than that resulting from the play of two cooperators. In the pioneering work of Nowak and May (1992), control over the growth of defectors can be exerted by the topology of the emerging structures, where a cluster of defectors brings no gain to society, thereby making it possible for a cooperator surrounded by cooperators to generate more profit for the whole of society than a defector at the frontier of a cluster of defectors. The condition *b* > 2 does not generate a social dilemma, because in this case, there is no social control over the spreading of defectors who, in due time, fill the entire network.

Game theory with behavioral constraints aims at maintaining a state of social dilemma, thereby preventing an unlimited expansion of either cooperators or defectors through mutual influence, of level ℱ on level 𝒮 and of level 𝒮 on level ℱ. The social dilemma condition is subsequently proven to keep the social network at the critical point, thus ensuring its adaptability and flexibility, and the condition of maximal efficiency for information transport.

### 5.2 The dynamics of level 𝒮

The dynamics of level 𝒮 rests on the basic concept of *criticality*, which is borrowed from physics and extended to complex networks. Each individual of a set of *N* individuals makes a decision between two possible choices, *ξ* = 1 and *ξ* = −1, under the influence of the choices made by the individuals linked to it. In the absence of interaction, the mean field

(6) $$x=\frac{\sum_{i=1}^{N}\;\xi_{i}}{N}$$

vanishes, because the probability of selecting *ξ* = 1 is equal to the probability of selecting *ξ* = −1. If the interaction strength is large enough to achieve the critical value *K_C_*, the mean field becomes either positive or negative. It is important to stress that this is a phase transition and the critical value *K_C_* depends on the network topology.

The sociological literature of the last few years (Golam, 2004; Hong and Strogatz, 2011; Masuda, 2013; Sîrbu et al., 2013) has devoted some attention to the action of *contrarians*. A contrarian is an individual, interacting with the individuals linked to it, who is inclined to make a decision opposite to the opinion of the majority of its neighbors. The quantitative effects of contrarians on the global decision depends on the kind of statistical prescription adopted to study the social process, this being a subject of scientific debate. Using the DMM prescription, the results are remarkably simple and are used to outline the nature of the game theory based on the criterion of rapid achievement of consensus. For simplicity, we assume that the defectors on level ℱ act as contrarians on the level 𝒮. The adoption of the ATA condition makes it possible for a simple analytical prediction to be made about the influence of the action of contrarians. The transition rate from state 1 to state 2 in the ATA condition is given by

(7) $$g_{12}=\frac{g}{2}\text{exp}\left(-K\Pi \right),$$

where Π = *p*_1_ − *p*_2_ with *p*_1_ and *p*_2_ denoting the fraction of elements in the states 1 and 2, respectively. The meaning of Eq. (7) is transparent. If there are more elements in state 1 than in state 2, the transition rate of a given element from 1 to 2 is decreased. Under the opposite conditions, the transition rate is increased, thereby making it possible for the network to achieve consensus. Of course, for the transition from state 2 to state 1 the reverse condition

(8) $$g_{21}=\frac{g}{2}\text{exp}\left(K\Pi \right)$$

applies.

We would like to remark that the exponential structure of the transition rate is chosen so as to make the critical behavior of this model fall into the Ising basin-of-attraction (Fraiman et al., 2009), which is well known to physicists. However, this restriction can be bypassed as shown by the model for the emergence of altruism of Section 2.

In the presence of contrarians, the elements are separated into contrarians and cooperators, and the transition rates for the cooperators are denoted by

(9) $$g_{12}^{\left(C\right)}=\frac{g}{2}\text{exp}\left(-K\Pi \right)$$

and

(10) $$g_{21}^{\left(C\right)}=\frac{g}{2}\text{exp}\left(K\Pi \right),$$

i.e., the prescriptions corresponding to Eqs (7) and (8), respectively. The cooperators make decisions according to the majority of the individuals. The contrarians (defectors), on the contrary, adopt

(11) $$g_{12}^{\left(D\right)}=\frac{g}{2}\text{exp}\left(K\Pi \right)$$

and

(12) $$g_{21}^{\left(D\right)}=\frac{g}{2}\text{exp}\left(-K\Pi \right).$$

The master equation leading the cooperator mean field, described by $\Pi_{C}=p_{1}^{\left(C\right)}-p_{2}^{\left(C\right)}$, reads

(13) $$\frac{d}{\mathrm{dt}}\Pi_{C}=\frac{g_{21}^{\left(C\right)}-g_{12}^{\left(C\right)}}{2}-\frac{g_{21}^{\left(C\right)}+g_{12}^{\left(C\right)}}{2}\Pi_{C}$$

and the master equation leading the dynamics of defector mean field, described by $\Pi_{D}=p_{1}^{\left(D\right)}-p_{2}^{\left(D\right)}$, reads

(14) $$\frac{d}{\mathrm{dt}}\Pi_{D}=\frac{g_{21}^{\left(D\right)}-g_{12}^{\left(D\right)}}{2}-\frac{g_{21}^{\left(D\right)}+g_{12}^{\left(D\right)}}{2}\Pi_{D}.$$

The two master equations yield the equilibrium conditions

(15) $$\Pi_{C}^{\mathrm{eq}}=\frac{g_{21}^{\left(C\right)}-g_{12}^{\left(C\right)}}{g_{21}^{\left(C\right)}+g_{12}^{\left(C\right)}}$$

and

(16) $$\Pi_{D}^{\mathrm{eq}}=\frac{g_{21}^{\left(D\right)}-g_{12}^{\left(D\right)}}{g_{21}^{\left(D\right)}+g_{12}^{\left(D\right)}}.$$

Noting that $g_{12}^{\left(D\right)}$ of Eq. (11) is identical to $g_{21}^{\left(C\right)}$ of Eq. (10) and that $g_{21}^{\left(D\right)}$ of Eq. (12) is identical to $g_{12}^{\left(C\right)}$ of Eq. (9), we obtain

(17) $$\Pi_{D}^{\mathrm{eq}}=-\Pi_{C}^{\mathrm{eq}}.$$

As pointed out earlier, we make the simplifying assumption that the contrarians and the congregators of level 𝒮 coincide with the defectors and the cooperators of level ℱ, respectively. Thus, the symbol *p_D_* denotes the fraction of contrarians and, when consensus is possible and *K* is sufficiently large, the global field reads

(18) $$\Pi^{\mathrm{eq}}=\left(1-p_{D}\right)\Pi_{C}^{\mathrm{eq}}+p_{D}\Pi_{D}^{\mathrm{eq}},$$

where $\Pi_{C}^{\mathrm{eq}}$ is the global field created by the cooperators and $\Pi_{D}^{\mathrm{eq}}$ is the global field created by the defectors. Thanks to Eq. (17), we turn Eq. (18) into

(19) $$\Pi^{\mathrm{eq}}=\left(1-2p_{D}\right)\Pi_{C}^{\mathrm{eq}}.$$

Eq. (15) can be written in the form

(20) $$\Pi_{C}^{\mathrm{eq}}=\tan\text{h}\left(K\Pi^{\mathrm{eq}}\right),$$

which, using Eq. (19), becomes

(21) $$\Pi_{C}^{\mathrm{eq}}=\tan\text{h}\left(K\left(1-2p_{D}\right)\Pi_{C}^{\mathrm{eq}}\right).$$

The transcendental equation generating the equilibrium mean field of defectors is the same as the transcendental equation generating the equilibrium mean field of cooperators. This apparently counter-intuitive property is the final result of 5 or 6 years of research by our group along the lines of the project (West, 2011). Also, although this result was derived independently of the recent psychological experiment (Kurt et al., 2014), both lead to the same conclusion. Kurt et al. (2014) found that the distribution of the time durations of the emotional states of two struggling people is characterized by the same deviation from an ordinary exponential distribution, regardless of whether the conflict is tractable or intractable, thereby implying in both cases the important role of memory (Bar-Tal, 2007; Kurt et al., 2014). This suggests that the neural dynamics activated by the struggle between two players obeys the same prescription, their emotional states being driven by complex systems, their brains, which share the same complexity.

**Figure 6:**
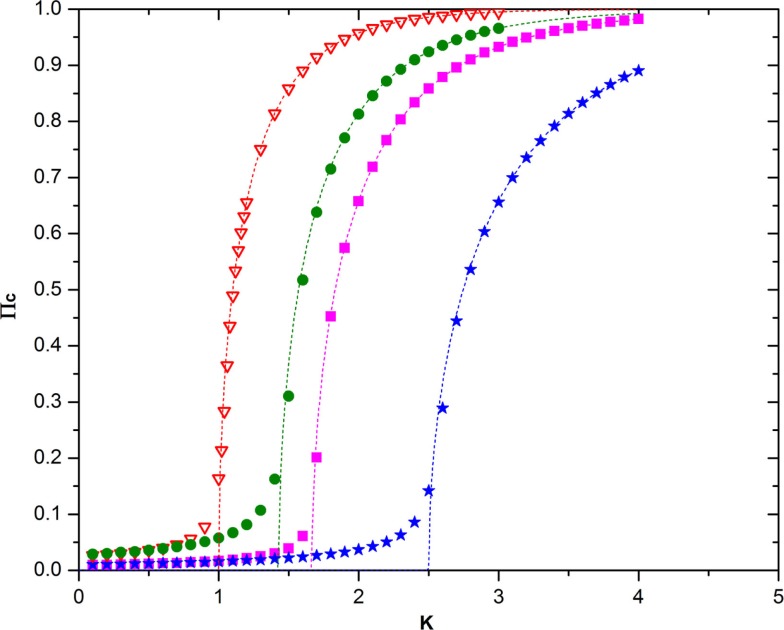
**This figure shows the remarkably good agreement between numerical calculation and the theoretical prediction on Eq. (21) in the ATA case**. The meaning of the symbols is as follows: red triangles = no defectors, *N* = 10^3^; green circles = 15% defectors, *N* = 10^4^, purple squares = 20% defectors, *N* = 10^4^; blue stars = 30% defectors, *N* = 10^4^, the dashed lines with the same color are the corresponding theoretical predictions.

**Figure 7:**
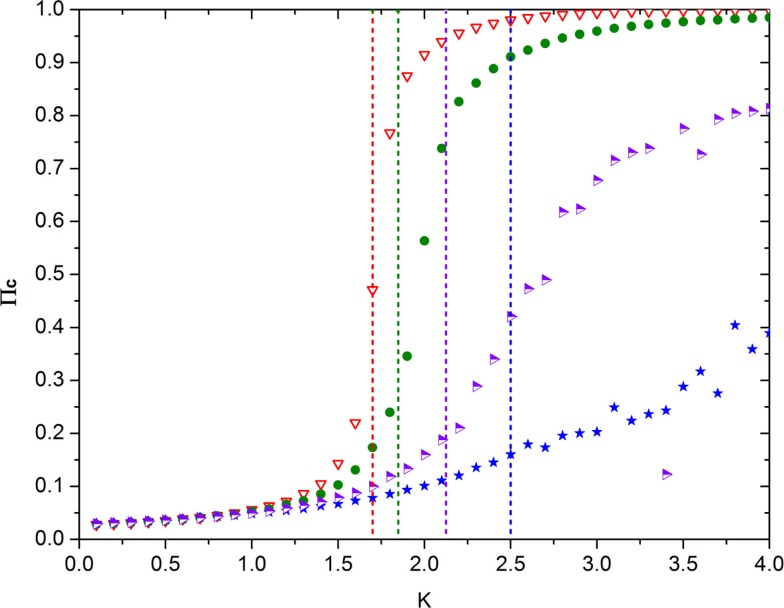
**This figure shows the emergence of the non-vanishing mean field upon increase of the control parameter *K* in the presence of different concentrations of defectors in the case of the regular two-dimensional lattice**. The meaning of the symbols is as follows: red triangles = no defectors, green circles = 4% defectors; purple-white triangles = 10% defectors; blue stars = 16% defectors. The vertical dashed lines denote the value of *K* corresponding to the earlier four increasing percentages of defectors (with colors matching that of the associated symbol). These straight lines have been obtained using Eq. (22) with *K_C_* = 1.7 and *χ* = 2.

Note that, in the ATA case, the prediction of Eq. (21) turns out to be in remarkably good agreement with the numerical results, as shown by Figure 6. Unfortunately, at the moment of writing this article, we do not have at our disposal an accurate analytical prediction for the case of a two-dimensional regular network, on which to play the new form of game theory. Figure 7 shows, however, that for a low concentration of defectors, the onset of criticality shifts to higher values of the control parameter that in this case, in the absence of defectors, occurs at about *K* = *K_C_* = 1.6. We therefore make the assumption that, in general, it is possible to adopt the following prescription

(22) $$K=\frac{K_{C}}{1-\chi p_{D}},$$

where *K_C_* denotes the critical value of the interaction strength *K* in the absence of defectors and *χ* depends on the sensitivity of the system to the concentration of defectors. The vertical dashed straight lines of Figure 7 aim to illustrate the shift of *K* at increasing percentages of defectors. They have been plotted using Eq. (22) with *K_C_* = 1.7 and *χ* = 2 for the purpose of showing how the critical value of *K* increases with increasing *p_D_*. They are merely heuristic and may be trusted only for very small percentages of defectors. In other words, in the two-dimensional case, the random distribution of a high percentage of defectors generates a complex behavior that would require an appropriate theory, still missing, and for high concentrations of defectors make the order parameter itself fluctuate, as shown in Figure 7. This interesting phenomenon is beyond the scope of this article, and for the numerical illustration of this form of game theory, we adopt for simplicity Eq. (22) with *K_C_* = 1 and *χ* = 2, which is valid only in the ATA case, but which serves well the purpose of affording a qualitatively correct illustration of this form of game theory.

The interaction intensity necessary to reach consensus becomes infinitely large when half of the units are defectors, and no consensus is reached with cooperation efforts of moderate intensity. For

(23) $$p_{D}\geq \frac{1}{\chi }=\frac{1}{2}.$$

consensus is not possible, regardless of the intensity of the cooperation effort.

The prescription of Eq. (22), emerging from our research work, although simple, reflects a complex and interesting sociological condition. The interaction strength *K_C_* depends on the topology of the network and predicts the emergence of criticality when there are no defectors. The presence of defectors is not necessarily negative. In fact, assigning to the social network an interaction strength larger than *K* given by Eq. (22) has the effect of generating a supercritical state, making the system insensitive to its environment and incapable of exchanging information with another identical social network (Vanni et al., 2011). To make possible for a complex network to drive another complex network, both networks should be at criticality. Our research has recently shown (Zare and Grigolini, 2013) that a few individuals of a network 𝒜 driven by a few individuals of a network ℬ, with both networks at criticality, establish a surprisingly accurate synchronization between 𝒜 and ℬ. This theoretical result affords an explanation of a recent experiment done at Duke University (Pais-Vieira et al., 2013) on the transmission of information from a rat brain ℬ to a rat brain 𝒜. A few electrodes implanted in the brain of rat ℬ transmit a signal to a few electrodes implanted in the brain of rat 𝒜 and induce a surprising correlation between the motion of the whiskers of rat 𝒜 and the motion of the whiskers of rat ℬ. This surprising synchronization is explained if the two brains operate at criticality.

**Figure 8:**
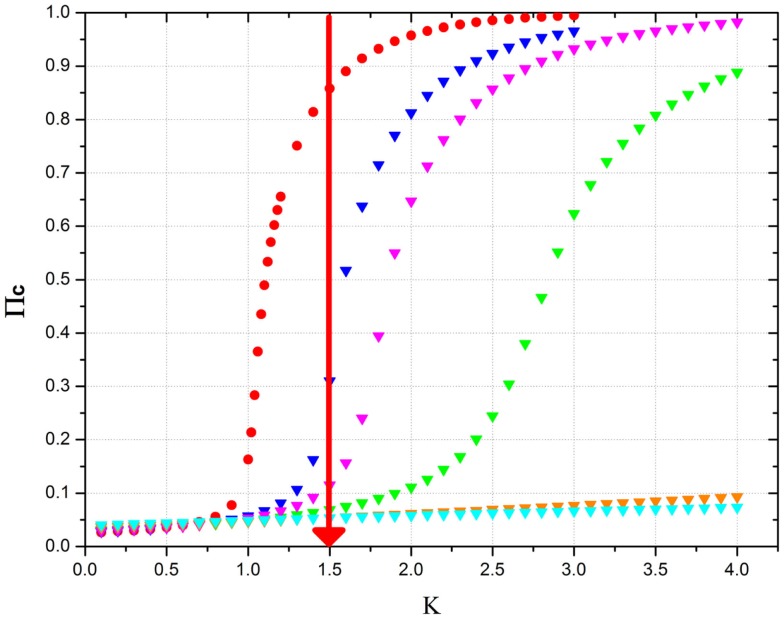
**This figure shows the equilibrium mean field generated by cooperators as a function of the cooperation strength *K* in the presence of an increasing percentage of defectors**. The meaning of the symbols is as follows: red circles = no defectors; blue triangles = 15% defectors; purple triangles = 20% defectors; green triangles = 30% defectors; orange triangles = 50% defectors; cyan triangles = 60% defectors. *N* = 10^3^. The time length of the program is 10^7^ time steps and the results have obtained by making averages on 10 realizations. The purpose of the red vertical line is an eye-guide showing which is the right percentage to reach to make the system critical.

Eq. (22) suggests that a sufficiently large concentration of defectors,

(24) $$p_{D}^{\left(\mathrm{crit}\right)}=\frac{1}{\chi }\left[1-\frac{K_{C}}{K}\right],$$

with *K* > *K_C_*, turns the supercritical into a critical condition, thereby rendering the social network sensitive to external stimuli and to the information generated by an identical social network. The beneficial role of defectors is illustrated by Figure 8. A large cooperation strength makes the network depart significantly from the critical condition, where the network is flexible and can adapt itself to new external conditions. The global field generated by cooperators yields a typical second-order phase transition. For values of the cooperation strength *K* smaller than the critical value established by the rule of Eq. (22), no consensus is possible and the cooperator’s global field vanishes at equilibrium. Figure 8 shows that increasing the percentage of defectors requires a higher and higher value of the cooperation strength *K* in order for the social network to reach consensus. In the absence of defectors, at a moderately large value of cooperation strength (*K* = 1.5 in the example of Figure 8, where *K_C_* = 1), the network is in a supercritical condition, inconvenient for the reception and transmission of information (Vanni et al., 2011). As an effect of the beneficial role of defectors, the system is shown to reach criticality when the defector percentage is about 15%, in accordance with the crucial percentage of Eq. (24), which is exact in the limiting case of infinitely many individuals. Figure 8 shows that the red line crosses the critical region between *p_D_* = 0.15 and *p_D_* = 0.20. A further increase of the defector percentage has the effect of shifting criticality to higher values of cooperation strength, thereby making the network, as indicated by the red line, fall into the sub-critical condition where no consensus is possible and the system becomes inefficient for information transport (Vanni et al., 2011).

**Figure 9:**
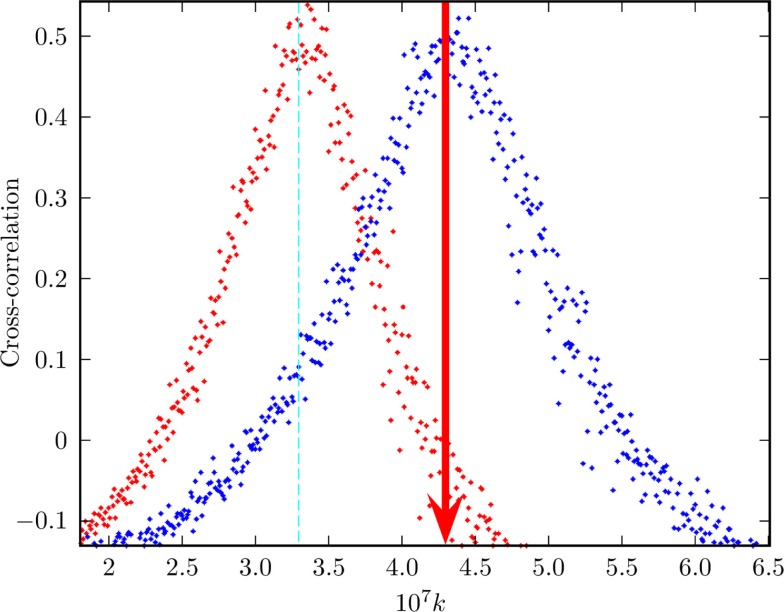
**Exchange of information between two neural networks**. This is the result of a numerical experiment measuring the transport of information from a network *B* (perturbing network) to a network *A* (perturbed network). Three percent of neurons of network *A* are forced to adopt the behavior of 3% of neurons of network *B*. The model adopted is the integrate and fire model of Usefie Mafahim et al. (2015) with random choice of initial condition replacing the stochastic force of Usefie Mafahim et al. (2015). The efficiency of the transport of information from *B* to *A* is evaluated by means of the correlation parameter *C*, discussed in Usefie Mafahim et al. (2015). The vertical red line is an eye-guide indicating the value of the cooperation strength necessary to reach criticality in the presence of 10% of inhibitory links.

The correspondence between the DMM’s properties and the neurophysiology of people debating contentious issues is not accidental. Inhibitory links play the same crucial role as contrarians in social systems. In the absence of inhibitory links, the neural network may be in a supercritical state. The exchange of information between two neural networks in this supercritical condition is very weak. Increasing the percentage of inhibitory links has the effect of recovering criticality and with it the maximal efficiency of information transport from one to another identical network. This interesting phenomenon is clearly illustrated by Figure 9, derived from the work of Usefie Mafahim et al. (2015). The transport of information between two neural networks with no inhibitory links and with the cooperation strength denoted by the red line of Figure 9 is very low. The action of 10% of inhibitory links has the effect of recovering the condition of maximal efficiency of information transport. This surprising similarity between social and neural networks sheds light into the social effects of behavioral psychology.

### 5.3 Interaction between level ℱ and level 𝒮

We have seen that the presence of defectors (contrarians) in the ATA condition has the effect of boosting the value of *K* necessary to reach criticality, according to the simple formula of Eq. (22), with *K_C_* = 1 and *χ* = 2. To use these results in the new game theory that we propose in this paper, it is necessary to adopt a two-dimensional regular lattice, where each player has four nearest neighbors, some of which may be defectors. In this case, we do not have yet at our disposal a simple formula as that of Eq. (22). We conjecture that a formula of the same kind, with *K_C_* ≠ 1 and *χ* ≠ 2 may be very close to the true solution. However, to illustrate with preliminary numerical calculations how the new game theory works, we use Eq. (22) with *K_C_* = 1 and *χ* = 2. The results produced by a more appropriate expression for *K* as a function of *p_D_* are expected to agree qualitatively with the numerical results of this subsection.

We adopt as control parameter *p_D_* rather than *K*. We assign to *K* a value that, in the absence of defectors, would correspond to the supercritical condition. Using Eq. (24) with *K_C_* = 1 and *χ* = 2, we obtain for *p_D_* the critical value

(25) $$p_{D}^{\left(\mathrm{crit}\right)}=\frac{1}{2}\left[1-\frac{1}{K}\right].$$

We select *K* = 2.1 and consequently, on the basis of Eq. (22), we obtain $p_{D}^{\left(\mathrm{crit}\right)}=0.262$. The game theory illustrated in this subsection will make the system evolve in such a way as to remain close to the critical condition $p_{D}^{\left(\mathrm{crit}\right)}=0.262$.

Let us imagine that the initial condition corresponds to $p_{D}>p_{D}^{\left(\mathrm{crit}\right)}$. In this case, there is no consensus rather there is a delay in making a decision, which is detrimental for society. The society must exert a control on the excessive growth in the number of defectors thereby preventing *p_D_* from going much beyond $p_{D}=p_{D}^{\left(\mathrm{crit}\right)}$. This control is exerted by assigning to the payoff *b* the value *b_C_* = 1.75, which is known (Nowak and May, 1992) to favor the increase of the concentration of cooperators. This is the control exerted on the growth of defectors on the basis of the observation that the lack of consensus corresponds to a societal financial disadvantage.

Now let us imagine that as a result of this control the concentration of defectors becomes very small, namely, $p_{D}<p_{D}^{\left(\mathrm{crit}\right)}$. In this condition, the sociological network becomes supercritical. This corresponds to the condition of a neural network when the number of inhibitory links becomes too small. In this case, as shown by Figure 9, the network is no longer flexible and is insensitive to the influence exerted on it by a neural network at criticality, due to the action of a sufficiently large number of defectors. In this condition, a sociological system obeying the DMM prescription would not be able to adapt itself to an unexpected emergency condition, as illustrated by the work of Vanni et al. (2011) with the example of a swarm of birds. The transmission of information from the environment to the system either becomes weak or is completely quenched (Vanni et al., 2011). To counterbalance this negative condition, the payoff *b* attains the value *b_D_* = 2.05 that according to Nowak and May (1992) would make the defectors fill the entire network. This has the effect of preventing significant penetration of the network into supercritical territory.

**Figure 10:**
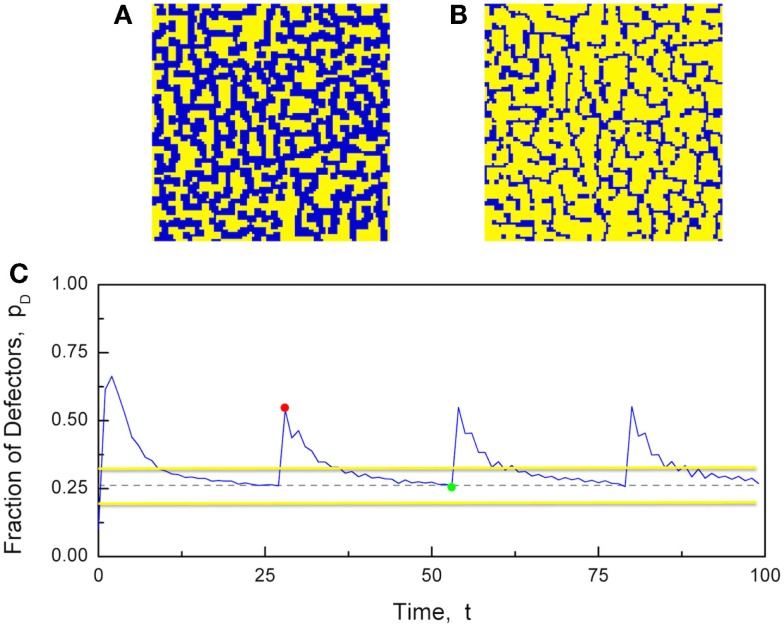
**The Nowak and May prisoner’s dilemma game, run on a 2D network with *N* = 100^2^ players, and modified to have two *b* values, *b_C_* = 1.75 and *b_D_* = 2.05**. The interaction strength was set to *K* = 2.1. **(A)** A snapshot of the strategies when there is a majority of defectors (blue). **(B)** A snapshot of the strategies when there is a majority of cooperators (yellow). **(C)** The time evolution of the fraction of defectors (blue line). The critical fraction of defectors is $p_{D}^{\left(\mathrm{crit}\right)}=0.262$ (dashed line). The snapshots in **(A,B)** were taken at times *t* = 29 (red dot) and *t* = 54 (green dot), respectively. The two horizontal yellow lines denote the stripe within which the social system remains at criticality, as a result of the finite-size effect (Hollingshad et al., 2013).

Figure 10 illustrates the numerical results generated by these prescriptions and shows that the desideratum of establishing consensus keeps the growth of defectors under control. As a consequence of this constraint, the fraction of defectors never reaches the maximal value *p_D_* = 1, corresponding to the complete defeat of cooperators. The concentration of defectors quickly moves toward a maximal value close to 0.5 followed by regression to the critical value $p_{D}^{\left(\mathrm{crit}\right)}$. The whole region $p_{D}>p_{D}^{\left(\mathrm{crit}\right)}$ corresponds to a vanishing degree of consensus. However, due to the finiteness of the number of elements, the fluctuations of the mean field in the critical region are very large and, as proved elsewhere (Hollingshad et al., 2013), criticality is not a singular condition limited to the dashed line of Figure 4, rather it applies to a relatively large stripe around the dashed line. Thus, the network spends a significant amount of time at criticality without penetrating the supercritical territory that would suppress its flexibility. We hope that these preliminary results may trigger the additional research work necessary to give a more solid foundation to this form of game theory. We note that the behavior illustrated by Figure 10 seems to be periodic and we conjecture that reducing the distance of *b_D_* from *b_C_* may have the effect of creating an intermittent process with the temporal complexity (Turalska et al., 2011) that was found to be a signature of criticality.

## 6 Cooperation-Induced Synchronization, Emergence of Cognition and Concluding Remarks

Recent work on the DMM sociological model (Turalska et al., 2012) suggests that cognition emerges as a result of criticality-induced long-range correlation. Herein, we use the neural and social theoretical perspective (Turalska et al., 2012) to develop a new form of game theory that mirrors the increasing conviction among behavioral psychologists that economic choices are not entirely “rational” (Shefrin, 2003; Shefrin and Statman, 2003). On the basis of the arguments illustrated by Rosenfeld (2013), that cooperation-induced locality breakdown is a form of Turing intelligence, we use the DMM theory (Turalska et al., 2012) to substantiate, through the statistical physics of phase transitions, the early hypothesis of McDougall (1928) concerning the existence of a collective mind, here rephrased as collective cognition.

### 6.1 Benefits and limits of the proposed forms of game theory

The separate individuals of the game theory proposed in Section 3 are individuals locally interacting according to the criterion of maximal financial benefit. The arguments that we use to constrain the growth of defectors match those adopted in the pioneering work of Nowak and May (1992, 1993). We supplement their theory by means of criticality-induced locality breakdown, which can be interpreted as global awareness of the benefits of cooperation. The excessive expansion of cooperators is counterbalanced by the super-criticality-induced loss of long-range perception, favoring the growth of defectors and with it the recovery of criticality and of a renewed perception of social benefit. The control of the excessive expansion of cooperators is an *ad hoc* property of this form of evolutionary game theory.

The form of game theory of Section 4 is not affected by the limits of this *ad hoc* condition. This is so because the cooperation strength *K* is fixed so as to maintain the level 𝒮 at criticality, and consequently in a condition of long-range perception. However, in this version of the game, the self-organization toward a criticality, moving from either a super- or a sub-critical condition, is lacking and it may be realized through a form of control such as feed-back.

### 6.2 Group mind, jung, kahneman, and tversky

The game theory proposed in Section 4 is expected to yield results even more ambitious than shedding light into the emergence of altruism, namely, to provide insight into the emergence of cognition.

According to Rosenfeld (2013), swarm intelligence (Vanni et al., 2011) is a form of Turing intelligence. This may lead to a reconsideration of the concept of group mind proposed in 1920 by McDougall (1928). In the first 20 pages of Chapter I of McDougall (1928), McDougall, replying to Maciver, another psychologist, writes: “the environment which influences the individual in his life as a member of an organized group is neither the sum of his fellow members as individuals, nor is it something that has other than a mental existence. It is the organized group as such, which exists only or chiefly in the persons of those composing it, but which does not exist in the mind of any one of them, and which operates upon each so powerfully just because it is something indefinitely greater, more powerful, more comprehensive than the mere sum of those individuals.”

American psychologists criticized McDougall because, in their opinion, mind is a property of a single (human) individual. Although the concept of group mind has been discarded by psychologists and cognitive scientists, there are good reasons to believe, as Van Bavel et al. (2014) point out, that the Aristotelian conviction of the highly social nature of human beings may well re-emerge shaping our beliefs about the origin of cognition.

We use the term cognition rather than mind, which to some may have religious implications, to maintain our research work at a rigorously scientific level. We can rephrase the criticism of the work of McDougall as the allegedly inappropriate assumption that a group of individuals may have cognition as a group rather than as single individuals.

This rephrasing of the criticism of McDougall’s work, however, opens the door to the problem of explaining the emergence of cognition in the brain of a single individual, which is unsolved and still closely related to religious beliefs about how the soul (mind) is connected to the brain. We note that the adoption of our interpretation of the work of McDougall, i.e., the assumption that cognition emerges at the social level, makes it possible to explain the origin of the collective unconscious of Jung (1991). The social group *S*, endowed with Turing intelligence (Rosenfeld, 2013), drives the level ℱ, the brain of a single individual, through the complexity management principle and this process can be interpreted as a social group transmitting its cognition to ℱ, thought of as the brain of a single individual.

It is interesting to notice that the transmission of cognition can be realized through the steady action of only a few individuals (Vanni et al., 2011; Luković et al., 2014), the parents of baby, on the brain of the baby, under the key assumption that the brain of the baby is a network at criticality, thereby in the proper condition to host cognition. This conjecture seems to be in line with recent advances in the field of neurophysiology (Pierce et al., 2014).

This is an ambitious but realistic hypothesis that may account for the transmission of the collective unconscious (Jung, 1991) from one generation to the next. We make the conjecture that the group mind has a conscious (group mind) and an unconscious (Jung) component, both of which are transmitted to single individuals through the complexity matching process advocated by our group as the optimal condition for the transfer of information from one network at criticality to another network at criticality (Zare and Grigolini, 2013).

On the one hand, according to Zafiovski (2014), the traditional view of economics, resting on the assumption that *homo economicus* makes fully rational choices based on the attainment of maximal individual benefit, is failing and researchers in economics are moving toward post-rational choice models involving (also) irrational or non-rational elements. On the other hand, the research work of Kahneman and Tversky on decision making under uncertainty (Shefrin and Statman, 2003) has triggered many psychophysics experiments that have contributed to establishing the connection between decision making and neural networks. This brings us back to the central idea of this article, that being the crucial role of criticality, and to the hypothesis that the brain is a network at criticality. For instance, the recent psychological experiment designed by Correll (2008) to disclose unconscious forms of racial prejudice has a neurophysiological foundation and the work (Grigolini et al., 2009) shows that the criticality hypothesis may help to obtain a deeper understanding of the results of this experiment. The growing networks of links between disciplines seem to obey the same cooperative processes as those hypothesized to be foundational for the emergence of intelligence (Villa Soto and Graf, 2013), thereby suggesting that the internet revolution may correspond to a new phase in the evolution of group mind (cognition).

### 6.3 Cognition and social cognition

In accordance with the title, our approach is expected to afford important contributions to improving the current knowledge about cognition. We would like to attract the attention of the readers to a few recent publications supporting our view. Werner (2011) recently discussed the widely accepted conviction about the close connection between computational science and intelligence and argued that it is nothing more than a metaphor. He explicitly wrote that his essay is equivalent to apply the Latin statement *sic transit gloria mundi* to Cybernetics. Werner wrote that recent experimental results in neurophysiology corroborate the idea of criticality and “violate the intents of the ‘framers’ of the Computational Metaphor for whom computation was discrete (and generally synchronous) in the programmable case, and continuous in Neural Networks.” Another aspect of the neural dynamics of the brain that the Computer Metaphor cannot successfully address is its unpredictability and its non-ergodic nature. In a more recent paper, published in a special issue of Chaos, Solitons and Fractals, devoted to *Emergent Critical Brain Dynamics*, Werner (2013) reiterated the statement that the brain does not compute and he quotes the paper (Grigolini et al., 2009) (earlier mentioned by us to explain the experimental observations of Correll) and other articles of our group as a paradigmatic example of cognition emergence. Temporal complexity is associated to the occurrence of renewal non-Poisson processes that are responsible for aging and ergodicity breaking. This is confirmed by the technique adopted by Fingelkurts et al. (2013) to detect rapid transitional periods. These authors found the brain to be in the exact condition necessary to produce the 1/*f* noise Grigolini et al. (2009). Another remarkable experimental observation supporting Werner’s theoretical perspective about cognition is that of Allegrini et al. (2013). These authors found that the transition from the unconsciousness of sleeping people to the awake state is to some extent equivalent to the transition from the sub-critical to the critical condition and to the related temporal complexity. Many authors, for instance Kitzbichler et al. (2009), emphasize the important role of criticality for information transport from one to another area of the brain, although the role of non-ergodicity in the information transport is not yet understood. We hope that this paper may contribute to the generation of a debate on this important aspect, and we plan in fact to expand in future work the non-ergodic theoretical arguments of Section 4.

Finally, to establish a connection with our arguments about Jung and the subconscious, we would like to mention the research currently made along the lines of the original work of Blanco (1988). This work, dealing with human cognition, addresses the important purpose of connecting cognition with emotion, a challenging issue that according to Werner cannot be settled by using the computer metaphor. In fact, some authors, for instance Lauro-Gotto (2008) are connecting the origin of emotions to the subconscious level that is described as a kind of network characterized by ultra-metric topology reminiscent of the topology of spin-glasses (Mezard et al., 1987). How does the subconscious network communicate with the conscious network? We make the conjecture that this may be done, thanks to the complexity matching and complexity management that play a fundamental role in the theory that we are proposing in this article. We want to stress that in our theoretical approach the nature of the cooperating units is not important, and this is the reason why, as emphasized by the title, we extend the emergence of cognition from the human mind to social networks, thereby going back to the view of McDougall but supporting his view with the crucial role of criticality. In the next section, we shall discuss further recent contributions to social cognition and their connection with the theory proposed in this article.

### 6.4 Looking ahead

We live in times of rapid progress in physiology as well as network science. The Frontiers special issue *Toward a Neuroscience of Social Interaction* (Pfeiffer et al., 2014) hosts 52 interesting articles, some of which either afford solid support to the apparently conjectural assumptions made in Section 2 or illustrate open problems that may be settled by research work done in the direction laid out in the present article. The neurophysiological roots of imitation by social interaction which are essential for the growth of altruism referred to in Section 2 and the game theory of Section 4 are discussed in detail by Froese et al. (2012) and Dumas et al. (2012). An overview of the most commonly employed economic games in social neuroscience (Engemann et al., 2012) supports our proposal regarding the interaction between the sociological level 𝒮 and the behavioral level ℱ. The research work (Coey et al., 2012) mentions the open problem of going beyond the limits of periodic behavior to study the correlation between different agents in a social context, an issue that may benefit from the complexity matching perspective we have advocated (Turalska et al., 2009; Zare and Grigolini, 2013; Luković et al., 2014) and which has been successfully adopted by Kello’s group (Abney et al., 2014) to study dyadic conversation and social interactions in general. The synchronization between the neural network *A* and neural network *B* of Figure 9 seems to be quite appropriate to account for the transmission of information from the right frontal area of the speaker to the medial frontal area of the listener illustrated in Figure 6 of Kuhlen et al. (2012).

The complexity matching process advocated herein can be used to afford the proper “non-reductionist” theoretical tool that the authors DiPaolo and Jaegher (2012) need to account for the coupling between the brains of two interacting members that cannot be established by observing their coordinated behavior. This latter article also reinforces the claim of Section 2 on the recent progress of neurophysiology supporting the group mind theory by McDougall. They (DiPaolo and Jaegher, 2012) attempt a mapping of: “the spectrum of possible relations between social interaction and neural processes” with a theoretical hypothesis where “social understanding happens in the absence of immediate interaction.”

The readers can find a further example of rapid interdisciplinary progress involving sociology and neurophysiology in the recent issue of Nature Neuroscience *Focus on social neuroscience*). The interesting articles of this issue give strong support to the herein proposed models, which rest on the actions of interacting units inspired by the actions of people. According to Nature Neuroscience, the “brains” of these units are shaped by nature so as to favor social behavior, and the statistical properties of the models of this article are compatible with this condition. As mentioned in this section, the model of Section 2 is based on the assumption that the altruistic choice made by some units is imitated by their neighbors, an indication of their social nature. Also, the decision making model (DMM) of Section 3 and Section 4 is based on the imitation assumption and consequently on the social nature of the interacting units. McCall and Singer (2012) illustrate the first steps that have recently been made in neuroendocrinology to shed light on affiliative behavior in humans, thanks to the rich information provided by the study of the social behavior of non-human animals. The review of Zaki and Ochsner (2012) shows the increasing interest researchers have in establishing the neural origin of empathy, so as to determine brain–behavior links. The articles of Eisenberger and Cole (2012) and Davidson and McEwen (2012) confirm the importance of social relationships for physical health, an important fact to take into account even if the social environment may be the source of psychiatric disorder (Meyer-Lindenberg and Tost, 2012). Although some important properties of social interaction revealed by these studies, for instance the neural origin of third-party punishment (Buckholtz and Marois, 2012), are not yet considered by the herein illustrated theoretical approach to a new form of game theory, we are convinced that such modeling can be properly extended to include them without weakening the importance of the connection between evolutionary game theory and criticality.

## 7 Conclusion

The most important element of novelty in this article is the connection of the new types of game theory with criticality. The main purpose of game theory is to establish the origin of altruism in sociological systems. In the literature on this subject, insufficient attention has been devoted to the fact that many emergent properties of organized societies must be the result of a phase transition from the units being virtually independent to being correlated over large distances in space and time. The properties at the phase transition, on the other hand, correspond to an out-of-equilibrium condition characterized by ergodicity breakdown. As a consequence, this article establishes a connection between socio-psychological processes and the ergodicity breakdown of anomalous diffusion in biological cells (Metzler et al., 2014).

Perc and Grigolini (2013) have challenged researchers in the field of evolutionary game theory to establish a connection with the neurophysiology of the brain, and especially with cognition, Hebbian learning and criticality-induced long-range correlation (Turalska et al., 2012). As shown in the latter reference, a deep similarity exists between neurophysiological and sociological processes, and for evolutionary game theory to fit the interdisciplinary trend of the evolving science of complexity, significant efforts are required to shed light on the origin of this similarity. This is equivalent to generating forms of game theory taking into account that behavioral psychology yields experimental evidence (Kearns, 2012) on the social nature of the human brain. The present article is the first salvo in the intellectual assault on the challenge raised by Perc and Grigolini (2013).

## Conflict of Interest Statement

The authors declare that the research was conducted in the absence of any commercial or financial relationships that could be construed as a potential conflict of interest.
